# GCEA-YOLO: An Enhanced YOLOv11-Based Network for Smoking Behavior Detection in Oilfield Operation Areas

**DOI:** 10.3390/s26010103

**Published:** 2025-12-23

**Authors:** Qing Liu, Xiaojing Wan, Yuzhou Sheng, Shuo Wang, Bo Wei

**Affiliations:** 1 School of Chemical Engineering, Xinjiang University, Urumqi 830049, China; 13999253675@163.com (Q.L.); 17615263893@163.com (B.W.); 2 School of Modern Industrial Engineering for Intelligent Manufacturing, Xinjiang University, Urumqi 830049, China; claouaywhyeyskusyahwywf@gmail.com (Y.S.); 15169701380@163.com (S.W.)

**Keywords:** deep learning, feature fusion, oilfield safety, smoking detection, YOLOv11n

## Abstract

Smoking in oilfield operation areas poses a severe risk of fire and explosion accidents, threatening production safety, workers’ lives, and the surrounding ecological environment. Such behavior represents a typical preventable unsafe human action. Detecting smoking behaviors among oilfield workers can fundamentally prevent such safety incidents. To address the challenges of low detection accuracy for small objects and frequent missed or false detections under extreme industrial environments, this paper proposes a GCEA-YOLO network based on YOLOv11 for smoking behavior detection. First, a CSP-EDLAN module is introduced to enhance fine-grained feature learning. Second, to reduce model complexity while preserving critical spatial information, an ADown module is incorporated. Third, an enhanced feature fusion module is integrated to achieve effective multiscale feature aggregation. Finally, an EfficientHead module is employed to generate high-precision and lightweight detection results. The experimental results demonstrate that, compared with YOLOv11n, GCEA-YOLO achieves improvements of 20.8% in precision, 6.9% in recall, and 15.1% in mean average precision (mAP). Overall, GCEA-YOLO significantly outperforms YOLOv11n.

## 1. Introduction

Oilfield smoking violation is a significant but avoidable cause for fire and explosion. Globally, open flame events (smoking violations included) have caused at least 50–70 deaths and over USD 2 billion in economic loss in the upstream oil and gas sector between 2015 and 2023 [1]. In China, there were approximately 80–90 smoking-related fire accidents in oil and gas fields from 2018 to 2024, causing around 10–15 deaths and direct losses exceeding CNY 180 million [2]. There are numerous flammable materials in oilfields, so even if there is a minor fire, it could lead to fires or explosions that cause significant casualties, environmental pollution, and equipment damage [3]. Fires and explosions happen because of insufficient safety compliance. Severe explosion accidents have happened all over the world; for instance, in 1998, a fuel tanker train explosion in Yaoundé, Cameroon, was triggered by leaked fuel vapors ignited by an open flame such as a dropped cigarette, resulting in more than 200 fatalities [4]. The causes of fires and explosions in oilfields are many. Among them, the accidents caused by the unsafe behavior of workers are avoidable and traceable [5]. Intelligent security is one of the components of an intelligent industrial system, and its development plays an important role in improving the safety management level of operation scenes [6]. With the arrival of the big data era, data-driven safety science is moving towards systematic and intelligent development [7]. This gradual development is clearly an important direction for promoting profound changes and constant improvements within the realm of intelligent security [8]. And also, it is necessary to use intelligent methods to deal with the dangers in oil fields caused by people who smoke [9]. 

Traditional detection methods mainly depend on smoke detectors, manual patrols, and manual identification through video surveillance [10]. Huang et al. [11] proposed a method to detect smoking behavior by using an SVM (support vector machine), which they used to model and classify smoke features and cigarette handling actions. However, it is overly reliant on movement and smoke features, which have certain limitations. Fu et al. [12] proposed an enhanced convolutional neural network design for real-time detection of smoking and mobile phone usage behavior, yet within complicated background settings, the model’s capacity to identify tiny targets proves to be rather limited.

In recent years, due to the rapid development of machine vision and image processing technology, many deep-learning-based methods have been proposed to detect violations in videos and images automatically [13]. These methods have important practical significance in fire safety management. Additionally, advances in hardware technologies, especially CPUs and GPUs, have made it possible to perform large-scale high-performance computing, enabling the processing of big data and training complex models. Deep learning can extract features directly from raw data, and this capability has led to significant advances in the field of object detection [14]. Neural-network-based methods have become the mainstream approach [15].

However, deep-learning-based algorithms still have significant challenges in a practical smoking detection application. Firstly, cigarettes belong to small objects with many detailed features that are difficult to detect accurately when it comes to lighting, background changes, and various holding postures, resulting in poor detection accuracy and inaccurate localization. Secondly, traditional FPNs tend to lose critical information when combining features at different scales, so they inadequately capture contextual information regarding smoking behavior [16]. Additionally, standard downsampling causes significant information loss on small objects such as cigarettes [17], and conventional detection heads have poor computational efficiency in dense scenes and are unable to satisfy the requirements of real-time monitoring systems [18]. 

To achieve accurate, real-time, automated prevention of smoking-induced fires in oilfields, we propose a GCEA-YOLO framework that greatly reduces manual inspection workload and improves on-site management efficiency. The main contributions of this paper are as follows:

1. This paper presents Adown, which is an attention-based downsampling module that uses average pooling, max pooling, and convolutional attention [19]. It retains important features of small objects but reduces their spatial resolution so that the model can still find them even when they are tiny.
2. We propose CSP-EDLAN (cross-stage partial enhanced dense local aggregation network), an enhanced dense connection module inspired by ELAN architecture [20]. By integrating dense connections [21] within CSP structures [22], CSP-EDLAN strengthens feature propagation and gradient flow, enabling better representation learning for small object detection while maintaining computational efficiency.
3. To improve detection speeds, we have created an efficient head detection module that uses lightweight group convolution [23] and a dual path of 3 × 3. With the addition of decoupled prediction branches [18], the EfficientHead mechanism requires low computation costs but still can obtain accurate detection results in resource-limited situations.
4. As compared to the present state-of-the-art detection models, the GCEA-YOLO framework has achieved better accuracy while maintaining high-speed inference by using a global feature pyramid network (GFPN) [16]. The GFPN uses CSPStage modules for multiscale deep feature learning, which allows for effective global context integration and makes it suitable for real-time smoking behavior detection and practical industrial applications.

On top of these modules, we present GCEA-YOLO, which is a framework tailored for small target detection in smoking behavior recognition. And it can also improve the feature extraction ability in complex situations, speed up the convergence rate, and improve the overall detection accuracy [24]. From experiments, we can see that GCEA-YOLO achieves 12.8% and 3.6% improvement with respect to precision and recall, respectively, using only 1.4 GFLOPS more than other YOLO models [25], making it highly practical for real-time edge deployment in oilfield environments. In this paper, the rest is organized as follows: Related works are reviewed in Section 2. The proposed modules and improvement methods are presented in Section 3. The experimental dataset, evaluation metrics, and results are presented in Section 4. Conclusions and Future Work are discussed in Section 5.

## 2. Related Work

### 2.1. General-Purpose Detection Networks and Architectural Limitations

Currently, mainstream object detection algorithms can mainly be classified into two types: two-stage detection methods and single-stage detection methods [26]. The two-stage detection algorithms with the R-CNN family as representatives [27] follow a process where it first generates some region proposals and then extracts features, classifies objects, and regresses bounding boxes to achieve precise object recognition. Faster R-CNN [28] and Mask R-CNN [29] models have strong detection capabilities in complex situations, but due to their multi-step processing pipelines and high computational requirements, they are difficult to use in real-time detection applications. On the contrary, single-stage detection algorithms such as YOLO [30], SSD [31], and RetinaNet [32] carry out object classification and localization directly on feature maps in an end-to-end fashion, which makes these models efficient and hence suitable for real-time scenarios. In recent years, the YOLO series has achieved great success by combining multiscale feature fusion, attention mechanism, and lightweight architecture design, improving detection accuracy, keeping fast inference speeds, and thus becoming the most popular and fastest developing algorithm framework in object detection [24]. YOLOv11 [33] is the eleventh version of the YOLO series and also one of the most advanced object detectors today.

But these general-purpose models cannot be implemented in actual smoking detection situations right away, as they mainly run into three big obstacles: (1) Oilfield environments have complicated and various backgrounds, which make traditional convolutional neural networks easily affected by environmental interference, resulting in poor recognition accuracy for smoking behavior [34]. (2) Cigarette targets in smoking detection have small sizes and elongated shapes, so consecutive downsampling operations in the network will lead to the substantial loss of small-target feature information [35], and at the same time, it is difficult to make full use of shallow fine-grained features. (3) Traditional convolutional neural networks have difficulty achieving balanced detection accuracy and computational speeds at the same time [36], so they do not work well enough for real-world needs that require fast and accurate detection.

Although each iteration of the YOLO series (v5–v12) has progressively reduced the model size and increased the average accuracy, these models still have three major architectural limitations when it comes to detecting smoking in oilfield environments:

- Aggressive strided convolutions or standard pooling in the backbone causes an irrecoverable loss of spatial detail for elongated cigarette objects, which occupy less than 2% of the pixels, This leads to lower detection precision on our challenging oilfield dataset.
- The conventional FPN/PANet-based necks have limited cross-scale interaction paths, which makes it difficult to retain both shallow hand textures and deep context clues needed for spotting cigarettes from 5 to 30 m away during sandstorms or poor visibility.
- Detection heads remain too computationally expensive or not sufficiently optimized to allow stable real-time inference on the explosion-proof edge camera, which has strict power and thermal constraints.

These persistent general-purpose lightweight detector problems account for why they do not perform well in real-life oilfield smoking violation scenarios, which is what makes the targeted architectural redesign suggested here necessary.

### 2.2. Review of Specialized Smoking Detection Models

Though such efforts have been made in recognizing smoking behavior, the current specialized models are still unable to overcome the three fundamental architectural limitations of general-purpose detectors (Section 2.1) in harsh oilfield environments: severe loss of fine-grained features of tiny elongated cigarettes during aggressive downsampling, insufficient cross-scale and cross-hierarchical feature interaction, and excessive computation in the detection head.

Table 1 explicitly links the most representative recent works to these three persistent bottlenecks. As shown in Table 1, none of the existing specialized models simultaneously address all three bottlenecks—most critically, the aggressive downsampling loss of tiny elongated cigarette features. The most representative recent works are briefly reviewed below, which show that these problems remain unsolved. Wang et al. [37] introduced the SD attention mechanism and a gradient distribution strategy that effectively balances multiscale fusion of spatial and semantic information by weighting and aggregating features from different positions. Wang et al. [38] proposed a content-aware feature reshaping module, CARAFE, which uses dynamically generated adaptive convolution kernels to adaptively rearrange multiscale features, greatly improving the detection performance of small targets. Zhang et al. [39] proposed ECA-FPN, which improves the semantic alignment between the intermediate and shallow layers and reduces the redundant computation in the fusion process of the feature; thus, it achieves a good balance between the lightweight model and detection performance.

Although there were some positive outcomes in certain circumstances, the aforementioned methods had a few restrictions as well. Firstly, they have weak cross-domain generalization ability, and their performance will drop sharply when the model is transferred to other industrial fields. Secondly, these methods lack sufficient discrimination for very tiny or densely occluded objects. Thirdly, the present upgrades mainly concentrate on local module enhancements without involving comprehensive cooperation between the backbone network and feature fusion to the detection head.

### 2.3. Attention Mechanisms

Attention mechanisms are an important part of deep learning, and they can make the network pay attention to some important areas and features by changing the weight of features and ignoring useless information so as to improve its ability to represent features and its detection accuracy. The widely used attention mechanism includes channel attention (SE module in SENet), spatial attention, self-attention mechanism (Transformer), hybrid attention (CBAM), and coordinate attention. In the field of object detection, many people have used an attention mechanism on the YOLO model and achieved good results.

Huang et al. [40] proposed SSA-YOLO, which improves the small-defect feature extraction ability by adding channel attention modules in the shallow layer of the backbone network, and they introduced the swin transformer’s self-attention mechanism into the neck network to enhance the multiscale defect detection performance. He et al. [41] used a channel purification method in DCGC-YOLO. This method allows the model to exchange information in the channel dimension via group convolution such that it can pay more attention to the important feature channels of flames and smoke. Huang et al. [42] used channel attention and feature distillation in YOLO-ULNet and maintained forest fire detection capability with reduced computation by pruning channels. Though there have been improvements, current attention mechanisms still have certain restrictions. High computational complexity makes it hard to achieve a good balance between accuracy and speed in lightweight situations; the ability to capture features and adaptively fuse small- and multiple-scale targets is inadequate, and some mechanisms prioritize local feature enhancement at the expense of global contexts and long-range dependencies.

## 3. Methods

### 3.1. Overall Architecture of GCEA-YOLO

**Figure 1:**
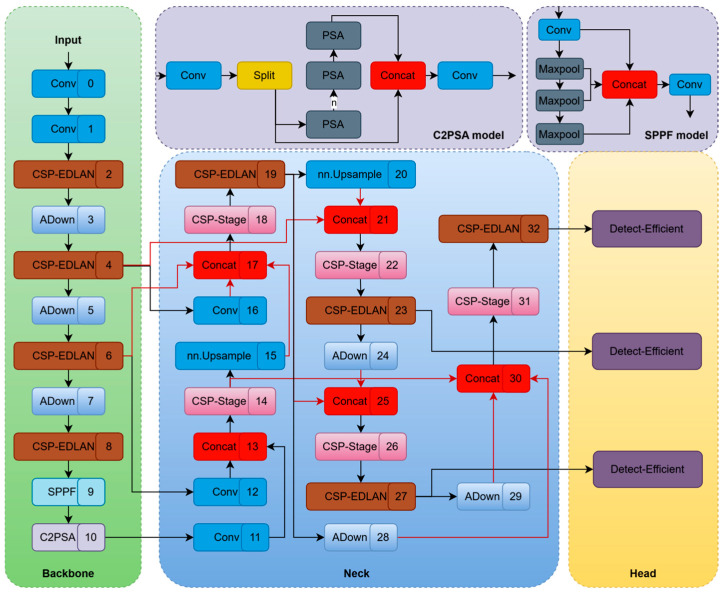
Overall structure of the GCEA-YOLO. Note: Numbers indicate the sequence of layers in the forward propagation path; modules with the same color represent identical structures.

Based on YOLOv11n, we present GCEA-YOLO, a high-precision and lightweight model for smoking behavior detection (Figure 1). In order to achieve high accuracy under edge computing constraints, we implement four main improvements. Firstly, we use the CSP-EDLAN module instead of C3k2 in the backbone, which can propagate features more richly without losing compactness due to cross-stage partial connections and DualConv (group + pointwise convolution), enhancing dense layer aggregation. Secondly, we use ADown modules to replace traditional pooling/strided convolutions, where adaptive downsampling keeps important features when reducing resolution so as to deal with scale changes and background noise [43]. Thirdly, we create a global feature pyramid network (GFPN) as a fundamental reconstruction of YOLOv11n’s simple feature fusion path. The original model uses upsampling and convolution for basic fusion, but GFPN forms complex multiscale bidirectional information flows [44]: At the P4 scale, 3-way fusion combines upsampled P5 features, downsampled P3 features, and original P4 backbone features; at the P3 scale, it fuses upsampled P4 features with original P3 backbone features to detect small objects; at the P5 scale, 3-way fusion merges initially processed P5 features with 2 downsampled paths from different P4 processing stages, achieving full cross-scale feature integration. After every fusion node, we adopt CSPStage for initial feature combination and CSP-EDLAN modules for deep feature improvement, along with dense connections that add even more richness to the features, thus allowing for the thorough fusion of low-level textures and high-level semantics via numerous pathways—providing much more discriminative multiscale representations compared to the original FPN. Lastly, we introduce a Detect_EfficientHead as a replacement for the traditional detect module. This utilizes group convolutions and lightweight branch networks [45] to greatly reduce the number of parameters and computation required without sacrificing accurate localization and classification performance, especially for small targets and occlusions [32]. These improvements make it possible for GCEA-YOLO to achieve the optimal balance of accuracy and efficiency for use at the edge in smart monitoring systems.

The overall network architecture is presented in Table 2 (core structure identical to Figure 1). Complete reproduction details, including loss functions, label assignment, and training settings, are provided in the bottom section of the same table.

### 3.2. CSP-EDLAN

In order to solve the problems of low computational efficiency, poor feature expression, and gradient flow in traditional object detection networks, this paper presents the CSP-EDLAN (cross-stage partial efficient dual-layer aggregation network) module. This module adopts the dual-path structure of DualConv, which combines grouped convolution and pointwise convolution to reduce computation while keeping good feature expression ability. The CSP structure can achieve efficient gradient propagation and feature reuse by means of multiple branches of dense connections.

**Figure 2:**
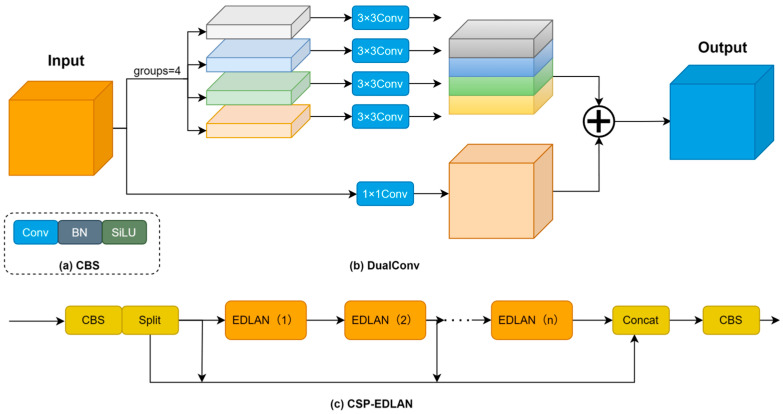
The specific architecture of CSP-EDLAN. (**a**) CBS; (**b**) DualConv; (**c**) CSP-EDLAN.

As can be seen from Figure 2c, this module employs a cross-stage partial design, where the input features experience channel transformation by the CBS layer (cv1) and then are divided equally into two branches, one of which will not be processed any further, while the other will be utilized for extracting features progressively by n stacked EDLAN units. All branch features (2 + n) are concatenated and fused using CBS (cv2) to create multiscale representations. As per Figure 2a, the CBS block has the following: convolution, batch normalization, and SiLU activation [46]. Figure 2b shows a dualconv structure with a parallel path; 3 × 3 grouped convolution (g = 4) captures local spatial features, 1 × 1 pointwise convolution allows cross-channel information exchange, and the outputs are combined by an elementwise sum. Each EDLAN unit has two DualConv layers. This design cuts down parameters by 1/g thanks to grouped convolution, yet it still ensures that cross-channel info flows via pointwise convolution. The CSP architecture’s multi-branch connectivity improves gradient propagation and feature reuse, so it can carry out real-time detection on resource-limited devices and also becomes stronger.

To quantitatively analyze the computational efficiency of CSP-EDLAN, we compare the FLOPs of DualConv with standard convolution. To quantitatively analyze the computational efficiency of CSP-EDLAN, we compare the FLOPs of DualConv with those of standard convolution. We define Do as the spatial dimension of the output feature map; *K* as the kernel size; *M* and *N* as the input and output channel numbers, respectively; *G* as the number of groups; *FL_SC_*, *FL_DC_*, and *FL_PC_* as the FLOPs of standard, grouped, and pointwise convolutions, respectively; *FL_ALL_* as the total FLOPs of DualConv (*FL_DC_* + *FL_PC_*); and *R_DC/SC_* as the computational efficiency ratio. Equation (1) defines the FLOPs of standard convolution. Equation (1) defines the FLOPs of standard convolution with a feature map size of *D_o_×D_o_*, kernel size of *K × K × M*, and *N* kernels. In DualConv, the features are divided into *G* groups, where grouped convolution (Equation (2)) and pointwise convolution (Equation (3)) operate in parallel. For the pointwise convolution branch, 1 × 1 kernels are employed (*K* = 1), which simplifies the standard convolution formula to (Equation (3)), where the *K^2^* term reduces to 1. The total FLOPs is given by Equation (4), and the computational efficiency ratio is derived in Equation (5). With *G* = 4 and *K* = 3, the ratio equals approximately 0.36, achieving 64% reduction in computational complexity while preserving feature representation. Our implementation employs *n* = 4 EDLAN units in the backbone and *n* = 2 in the detection head, balancing efficiency and accuracy for real-time edge deployment:

(1) $${FL}_{SC}=D_{o}^{2}\times K^{2}\times M\times N$$

(2) $${FL}_{DC}=D_{o}^{2}\times K^{2}\times M/G\times N/G\times G$$

(3) $${FL}_{PC}=D_{o}^{2}\times 1^{2}\times M\times N=D_{o}^{2}\times M\times N$$

(4) $${FL}_{ALL}={FL}_{DC}+{FL}_{PC}$$

(5) $$R_{DC/SC}=\frac{{FL}_{ALL}}{{FL}_{SC}}=\frac{1}{G}+\frac{1}{K^{2}}$$

### 3.3. ADown

**Figure 3:**
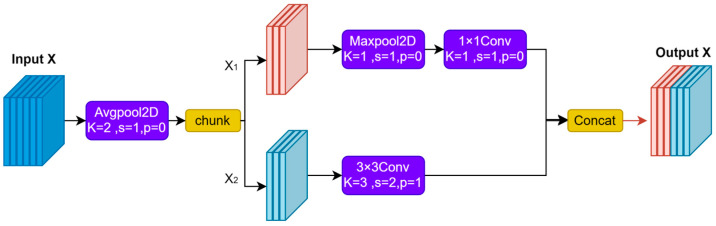
The specific architecture of ADown.

To address the problem of potentially losing important information when reducing resolution in smoking detection, we employ the ADown module [47] as a downsampling strategy, achieving good performance without excessive computational resources, as shown in Figure 3. ADown was originally proposed by Wang et al. for lightweight object detection, introducing a dual-branch collaborative feature compression architecture that effectively reduces computational cost while preserving important feature details. In our implementation, ADown replaces the traditional stride-2 convolutional downsampling layers in the YOLOv11 backbone. Its dual-branch design is particularly effective at preserving the spatial details and edge features of elongated cigar-shaped objects.

ADown employs a dual-branch fusion structure. First, the input features are gently pooled using average pooling to avoid losing too much information. Then, it splits into two halves along the channel dimension. The first branch uses 3 × 3 convolutions to keep spatial details and textures for long objects. The second branch uses 3 × 3 max pooling and then 1 × 1 conv to keep prominent edges and overall shape. Both branches are concatenated along the channel dimension. This design takes advantage of the detailed preservation of the convolution operation combined with the discriminative benefits of pooling, avoiding single-path information loss and keeping computational efficiency through channel splitting. Dual-path architecture provides multiple gradient flow paths during training, making it easier to train and effective at detecting small objects in images.

In order to demonstrate the efficiency of the ADown module quantitatively, we analyze the number of parameters and computational complexity. For an input feature map with a dimension of 160 × 160, the downsampled feature map will have a dimension of 80 × 80. We define *c* as the number of channels; *h* and *w* as the height and width of the feature map, respectively; *P_a_* and *F_a_* as the number of parameters and FLOPs of the ADown module; and *P_c_* and *F_c_* as the number of parameters and FLOPs of a standard 3 × 3 convolution with stride 2, which can be calculated by the following:

(6) $$P_{a}=\frac{5}{2}c^{2}$$

(7) $$F_{a}=\frac{5}{8}c^{2}\times h\times w$$

(8) $$P_{c}=9c^{2}$$

(9) $$F_{c}=\frac{9}{4}c^{2}\times h\times w$$

The constants derive from the following architecture: 5/2 comes from channel-split 3 × 3 and 1 × 1 convolutions (9*c*^2^/2 + *c*^2^/2); 5/8 accounts for spatial downsampling to *h*/2 × *w*/2; 9 equals the kernel size 3^2^; 9/4 includes the 1/4 downsampling factor. Based on the analysis of Equations (6)–(9), we can see that, compared with the traditional stride-2 downsampling convolution, the parameter quantity and computational cost of the ADown module are reduced by approximately 72%. The better performance of the ADown module is due to its dual-branch complementarity design: Depth-separable convolution retains much spatial detail information; the max-pooling path captures meaningful edge information. The two paths are fused together so as to solve the problem of feature degradation [48]. With the help of structural reparameterization [49], the ADown module keeps its multiscale detection ability, cuts down model complexity, and reaches a good mix of accuracy and efficiency for lightweight object detection work.

### 3.4. GFPN

**Figure 4:**
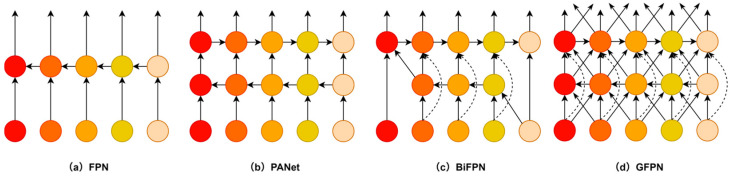
The specific architecture of GFPN. (**a**) FPN; (**b**) PANet; (**c**) BiFPN; (**d**) GFPN. Note: Different colors represent feature maps at different scales; solid arrows indicate feature flow; dashed lines denote bidirectional, repeated, or weighted connections.

Feature fusion is the main method of solving the problem of multiscale differences in object detection. The representative algorithms include FPN [16], PANet [45], BiFPN [50], etc., as shown in Figure 4a–c. These feature pyramid architectures gather multiscale features from the backbone network, but earlier ones only look at feature scales without considering hierarchy. To address this limitation and better detect cigarettes with multiscale characteristics in smoking behavior detection, we adopt the generalized feature pyramid network (GFPN) proposed by Tang et al. [51]. GFPN introduces a new kind of cross-scale fusion method that has both top–down and bottom–up pathways that interact bidirectionally. It employs MSCF, which takes what came out of the last node and puts it together with features from all sorts of scales and levels, letting those different-sized bits of information mix and talk to each other more fully, as we can see in Figure 4d.

**Figure 5:**
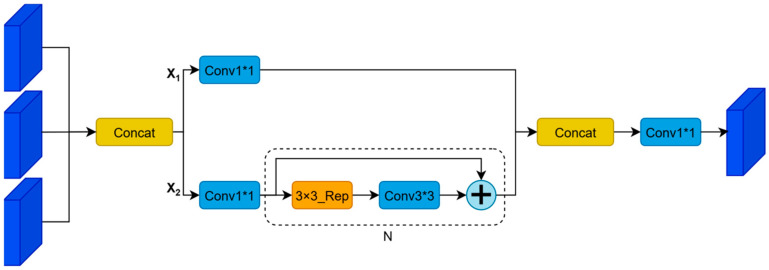
The specific architecture of CSPStage. Note: Different colors represent different module types; solid arrows indicate data flow; the dashed box denotes the repeated structure (N times).

From Figure 5, we can see that the architecture uses the CSPStage module for feature fusion. It divides the input feature into two streams by 1 × 1 convolution: one of them is the feature directly retained as X_1_, and the other one is extracted by n BasicBlock_3 × 3 _Reverse modules with inverted residual structure [52] and shortcut connection [49]. And then X1 and all intermediate features are concatenated together and passed through a 1 × 1 convolution to obtain the final fused feature. In this manner, it avoids the vanishing gradient problem [36] and allows gradients to flow to the deeper layers. GFPN integrates the MSCF mechanism with CSPStage’s dense fusion ability so as to achieve thorough cross-scale and cross-hierarchical feature interaction, yet it still controls the network’s depth, making it far better at utilizing global information than traditional unidirectional or bidirectional feature pyramid networks.

### 3.5. EfficientHead

**Figure 6:**
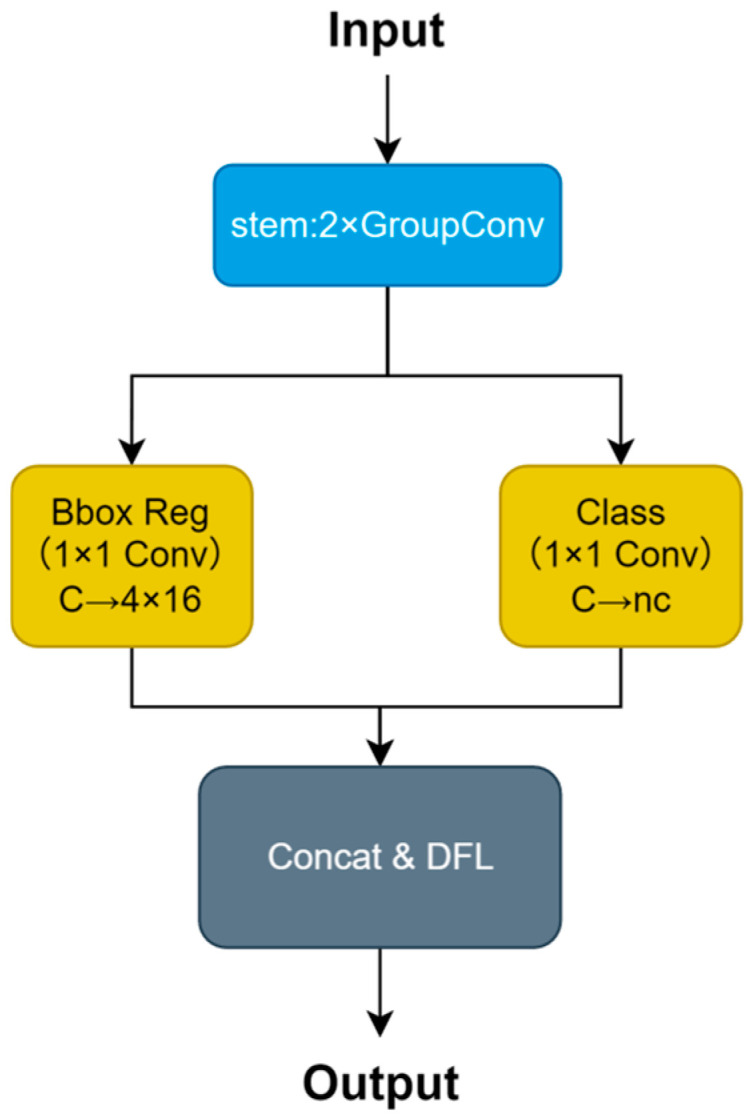
The specific architecture of EfficientHead. Note: Different colors represent different module types; solid arrows indicate data flow.

In order to solve the problems of computational redundancy and the presence of too many parameters in traditional YOLO detection heads, which affect real-time detection on edge devices when performing smoking detection tasks, this paper proposes an EfficientHead module with a lightweight design strategy consisting of three main parts. Unlike previous lightweight heads that mainly adopt GSConv [53], GhostConv [54], or shared convolution stems [55], EfficientHead introduces three distinct yet cooperative designs that have not been jointly used before: Firstly, two consecutive 3 × 3 group convolutions with g = c//16 (instead of commonly used g = 4 or 8) are used in the stem layer, greatly reducing computational cost. Secondly, there are two parallel 1 × 1 convolutional branches that separately carry out bounding box regression (producing 64-dimensional feature outputs) and class prediction, removing the need for multiple stacked layers of convolutions. Finally, the output layer changes the single point prediction of bounding box regression into a distribution prediction using concat operations and distribution focal loss [53], which is first combined with the above 1/16 ratio dual group–conv stem. An efficient pipeline is achieved with significant reductions in the number of parameters and computational complexity while still maintaining good enough detection accuracy, enabling better real-time performance and lower resource usage for deployment on surveillance cameras or nearby devices (Figure 6).

## 4. Experiment

### 4.1. Implementation Details

The experiment employed a constant input size of 640 × 640 pixels, and model training utilized SGD [56] as the optimizer. The initial learning rate started from 0.01 and then decreased to 0.0001 according to the cosine annealing policy [57], and the momentum coefficient was set to 0.937. In order to achieve a balance between model convergence and computation efficiency, training lasted for 200 epochs, with each batch containing 32 samples. All baselines and our proposed model are trained on the same hyperparameter setting, and no pretrained weights are used to initialize any of them for fair comparison. The exact software and hardware configuration used for the experiment is given in detail in Table 3.

### 4.2. Dataset

We created a new dataset called the Multi-Angle Smoking Detection Dataset, and it has 4847 smoking behavior pictures gathered from public places. Different complicated situations, such as sandstorms and hazy weather, are included so that the model can be more adaptable. We used LabelImg for image annotation and saved the annotations in XML format. Then, we converted them into YOLOv11-compatible txt files through a script. The dataset was split into training, validation, and test sets at an 8:1:1 ratio. In order to improve the robustness of our model, we applied different combinations of data augmentation techniques randomly on the training set. These techniques include horizontal/vertical flip, non-uniform scale, random shift, perspective transform, and random crop. All experiments were performed with training from scratch; no pre-trained weight was used. The dataset employs a single-class detection framework (nc = 1), with the class label “Smoke”. We define the “Smoke” class as the localized region where active smoking occurs, specifically encompassing the cigarette and the fingers holding it. The bounding box annotation focuses on this critical region rather than the entire person, enabling more precise detection while reducing false positives. Annotations were created using LabelImg and saved as XML files; then, they were converted to YOLOv11-compatible txt files with normalized coordinates [class_id, x_center, y_center, width, height], where class_id = 0 represents the “Smoke” class.

Dataset Features and Challenges: To give a full view of how complex the dataset is, we examine the main characteristics. All pictures have been resized to 640 × 640 pixels so that they can be used as inputs for the model. The dataset contains smoking behavior captured at different distances ranging from 5 to 30 m, with a focus on closer ranges (5–10 m), which are typical operating areas for workers in oilfield settings. The environmental conditions are varied. There are sunny days, smoggy days, sandstorms, and rainy or foggy days. Lighting conditions range from natural sunlight to low light during dawn/dusk and artificial lighting at night. The dataset has many issues, such as partial occlusion caused by objects, buildings, or other people, as well as crowded scenes with many people and person-to-person occlusions. Camera perspectives include front-facing views, side views, and elevated surveillance angles to capture a variety of monitoring scenarios. These features make this dataset representative of real-world oilfield surveillance situations and suitable for testing robust detection models.

### 4.3. Evaluation Metrics

The experiment employs some key indicators of object detection [58]. Precision (P) is the ratio of the number of true positive predictions to the total number of positive predictions (Equation (10)), while recall (R) is the ratio of the number of true positive predictions to the sum of the number of true positive predictions and the number of false negative predictions (Equation (11)), where TP, FP, and FN represent true positives, false positives, and false negatives, respectively. Average precision (AP) is computed by finding the area under the precision–recall curve using interpolation; a higher AP indicates better detection results. The mean average precision (mAP) is obtained by taking the average of AP over all categories, as given in Equation (12), where N is the total number of categories, and AP_i_ represents the average precision for the i-th category. Specifically, mAP@0.50 assesses the most basic localization performance at a single IoU threshold of 0.5, where a detection is considered correct if the overlap between the predicted and ground truth bounding box is greater than 50%. On the contrary, mAP@0.50:0.95 [59] provides a full picture of localization robustness by averaging mAPs calculated at 10 IoU thresholds from 0.5 to 0.95 with steps of 0.05 (Equation (13)), where j represents the IoU threshold value at 0.05 intervals, and mAP_(IOU = *j*) denotes the mAP calculated at threshold *j*. Also, it is evaluated by FPS (frames per second), which means how fast it can process images in real time, and Params, which means how many parameters the model has, showing how complex the model is:

(10) $$P=\frac{TP}{TP+FP}$$

(11) $$R=\frac{TP}{TP+FN}$$

(12) $$mAP@0.50=\frac{1}{N}\sum_{i=1}^{N}{AP}_{i}$$

(13) $$mAP@0.50:0.95=\frac{1}{N}\sum_{j=0.50}^{0.95}{mAP}_{IOU=j}$$

### 4.4. Ablation Studies

**Figure 7:**
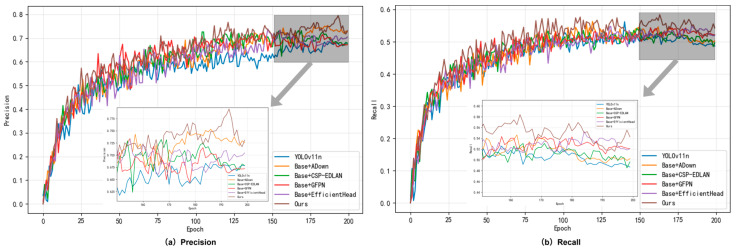
Training curves of precision and recall for ablation experiments.

To verify if every single part was effective, we conducted ablation studies [60] on YOLOv11n by starting from no module and adding them one at a time. As can be seen from Table 4 and Figure 7, performance improves progressively with each module added.

Baseline + ADown: Incorporating ADown independently enhanced precision from 0.614 to 0.701 (+14.2%) and also improved mAP@0.5 by 1.4% and mAP@0.5:0.95 by 2.8%. It notably reduced parameters by 19.23%, which demonstrated dual benefits for feature selection and model compression.

Baseline + CSP-EDLAN: This module significantly enhanced feature representation. It achieved 6.8% improvement on precision, 4.7% improvement on mAP@0.5, and 6.4% improvement on mAP@0.5:0.95 without sacrificing speed. The model maintained a high inference speed of 259.0 FPS due to the efficient reuse of features using dense connections.

Baseline + EfficientHead: EfficientHead enhances precision with only a small number of additional parameters by 5.7%, and recall was enhanced by 2.3%, which demonstrates that lightweight detection heads can effectively detect objects without introducing excessive complexity.

Baseline + GFPN: GFPN excelled at fusing features from different scales, improving mAP@0.5:0.95 by 9.6% and mAP@0.5 by 5.5%. Although there was an increase in computational cost, it proved to be highly effective for small objects and multiscale detection.

To investigate how modules interact and work together, we systematically analyzed representative pairs and groups of two and three modules. In terms of dual-module configuration, ADown + CSP-EDLAN demonstrated superior synergy between efficient downsampling and enhanced feature learning. It achieved a precision of 0.703 and mAP@0.5 of 0.543 with only 2.0M parameters, representing a 14.5% improvement on precision. ADown + EfficientHead achieved optimal compression at 1.8M params and 30.8% reduction, yet it maintained sufficient accuracy for demonstrating that lightweight designs were feasible. Notably, EfficientHead + GFPN attained the highest precision at 0.741 but a lower recall at 0.465, which indicates a bias towards precision over recall. For the triple-module configuration, ADown + CSP-EDLAN + EfficientHead achieved an optimal balance, with a precision of 0.711, a recall of 0.545, and 249.3 FPS, demonstrating an effective combination of lightweight design and detection capabilities. ADown + CSP-EDLAN + GFPN yielded the highest mAP@0.5 of 0.569 among the three-module combinations, which was 11.6% higher compared to CSP-EDLAN + EfficientHead + GFPN without ADown, requiring substantially more computational resources with 9.1 FLOPs and 3.9M params and confirming ADown’s importance for model efficiency. These ablation results provide three significant insights: First, ADown consistently reduces the number of parameters without compromising accuracy, thereby enabling more compact models that maintain performance. Second, GFPN appears in most combinations that achieve the greatest improvement in mAP@0.5:0.95, despite its higher computational cost. Third, the configurations based on CSP-EDLAN maintain the highest inference speed, with CSP-EDLAN + EfficientHead reaching 274.3 FPS, which enables real-time applications.

Synergistic Effect Analysis: Full integration achieved the best result with precision at 0.742, recall at 0.555, mAP@0.5 at 0.587, and mAP@0.5:0.95 at 0.255, which improved by 20.8%, 6.9%, 15.1%, and 17.0% compared to the baseline. Most importantly, it demonstrates that the whole model is superior to every combination of parts, which indicates that the different parts work collaboratively instead of interfering with one another [48]. With 3.4M parameters and 181.9 FPS, it achieves an optimal trade-off among accuracy, efficiency, and real-time performance. Ablation studies quantitatively validate both the effectiveness of individual modules and the synergies between them, thus validating the choice of fusion architecture for the object detection task.

Statistical Validation: To ensure reliability, each configuration was evaluated over three independent runs with different random seeds (29,11,2025). All experiments converged successfully with standard deviations below 0.01 for all metrics, demonstrating stable performance. The 95% confidence intervals for relative improvements of the full model over baseline are as follows: precision [+20.0%, +21.6%], mAP@0.5 [+14.4%, +15.8%], and mAP@0.5:0.95 [+16.2%, +17.8%], all entirely above zero (*p* < 0.001). No training failures or significant outliers were observed across 48 total experimental runs (16 configurations × 3 repetitions), confirming the robustness and reproducibility of our results.

### 4.5. Downsampling Module Comparison

To verify whether ADown was superior to other downsampling modules, we conducted a comparison experiment on the downsampling module. In the GCEA-YOLO model, we compared the performance of six different downsampling modules, which are SPD-Conv, MixDown, HWD, LDConv, ContextGuidedDown, and ADown. From Table 5, we can observe that the ADown module has the optimal overall accuracy with *p* = 0.742, R = 0.555, mAP@0.5 = 0.587, and mAP@0.5:0.95 = 0.255, and it requires only 7.8GFLOPs of computation and 3.4M parameters, which is the lowest computational cost compared to all other methods.

Compared to SPDConv with the second highest precision, ADown improves precision by 1.6%, recall by 10.1%, mAP@0.5 by 5.6%, and mAP@0.5:0.95 by 9.9%, and it reduces computational cost and parameters by 43.5% and 48.5%, respectively. Compared with MixDown with the second highest mAP@0.5:0.95, ADown has 3.7% improvement. Compared to the lightweight LDConv, ADown improves on mAP@0.5:0.95 by as much as 60.4%, demonstrating the optimal balance of accuracy vs. efficiency. From the experiments, we can observe that ADown achieves the best detection result with the lowest computational cost, confirming its superior and effective performance in the lightweight object detection task.

**Figure 8:**
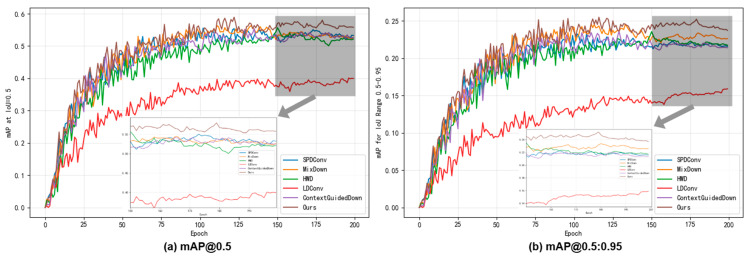
Comparison experiments of different downsampling modules. (**a**) the convergence curve of the mAP@0.5 metrics; (**b**) the convergence curve of the mAP@0.5:0.95 metrics.

From Figure 8, we can observe that this paper conducts a comparison of six different downsampling modules over 200 training epochs. Figure 8a,b show the convergence curve of the mAP@0.5 and mAP@0.5:0.95 metrics, respectively. The experimental results demonstrate that the proposed ADown module has superior overall performance during the whole training process. It not only converges faster than other methods but also achieves significantly higher final accuracy. On the contrary, the performance curves of the SPDConv, MixDown, HWD, LDConv, and ContextGuidedDown modules are still relatively low, and the ContextGuidedDown module has the worst detection accuracy. This visual analysis clearly demonstrates the complete superiority of the ADown module in terms of convergence behavior, training stability, and final performance. This confirms that it works effectively for lightweight object detection.

### 4.6. Comparison Experiments

In order to fully evaluate GCEA-YOLO’s capabilities, we conducted comparisons with the latest detectors that include two-stage methods, such as Faster R-CNN; single-stage detectors, such as SSD; and YOLO series models, such as YOLOv3-tiny, YOLOv5, YOLOv8, YOLOv10n, YOLOv11n, and YOLOv12n, as shown in Table 6. In terms of precision and recall under identical experimental conditions, GCEA-YOLO demonstrates obvious superiority: It achieves 0.742 for precision and 0.555 for recall, surpassing all compared YOLO-series models in both metrics. Not to mention, GCEA-YOLO achieves an mAP@0.5 of 0.587, which is significantly better than the best YOLO variant at 0.516. It also achieves an mAP@0.5:0.95 of 0.255, which is 17% higher than the YOLOv11n baseline. More importantly, as observed in Table 6, GCEA-YOLO has superior detection accuracy and is also efficient. With just 3.4M parameters and 7.8 GFLOPs in computational cost, it has 88% less parameters than Faster R-CNN, but it still achieves 181.9 FPS inference speed, satisfying the real-time requirement.

The results demonstrate that using CSP-EDLAN, ADown, EfficientHead, and GFPN modules works effectively, which indicates that our fusion strategy achieves superior performance for smoking detection. It provides an optimal balance of accuracy and speed for real-world applications.

### 4.7. Generalizability Validation on Public Dataset

Model generalization refers to the capability of a machine learning model to perform well on previously unseen data. This is crucial when we want to determine if a model can be used in practice. Strong generalization is essential to improve detection accuracy, avoid overfitting, and guarantee reliability in actual deployment.

To verify the generalization performance of GCEA-YOLO, we conducted thorough assessments using the Smoking Detection Dataset, which comprises 18,230 high-quality annotated images divided into training (16,758 images, 91.9%), validation (740 images, 4.1%), and testing sets (732 images, 4.0%). As shown in Table 7, we compared GCEA-YOLO with some state-of-the-art detectors such as SSD, YOLOv3, YOLOv5, YOLOv8, YOLOv10, YOLOv12, and Faster R-CNN. From experimental results, it can be observed that GCEA-YOLO has superior overall performance compared to other models, with mAP@50 at 88.8%, precision at 88.3%, and recall at 79.6%. Our method stands out by achieving an optimal balance between computational efficiency, model complexity, and detection accuracy, thus confirming the remarkable generalization ability of the proposed fusion structure.

To validate the generalization ability of GCEA-YOLO, we conducted comparative experiments using public datasets. The experiments used the COCO2017 dataset [62]. Because of the lack of computing power, we chose 8000 pictures as the training set, 1000 pictures as the validation set, and 1000 pictures as the testing set at random. GCEA-YOLO was compared to the basic model.

**Figure 9:**
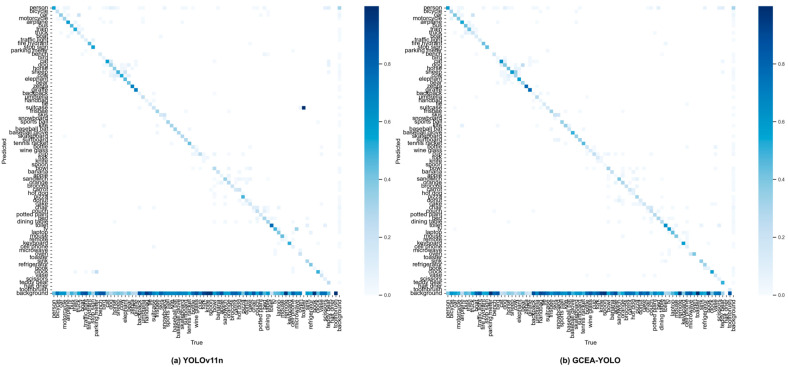
Normalized confusion matrices comparing detection performance on the COCO2017 validation set: (**a**) baseline YOLOv11n and (**b**) our proposed GCEA-YOLO model.

YOLOv11n: From Table 8, we can observe that, from the experimental results, GCEA-YOLO achieved better results than YOLOv11n in every metric: mAP@50 = 32.6%; precision = 45.9%; recall = 32.4%. As shown in Figure 9, the normalized confusion matrix provides details about each class’s detection performance. The increased diagonal intensity in Figure 9b indicates that GCEA-YOLO has higher classification accuracy over 80 object classes than YOLOv11n, which is shown in Figure 9a. Also noteworthy is that there is less confusion between classes in our model, especially those that are visually alike, such as cat/dog and car/truck, and fewer instances of backgrounds being classified as objects. This visualization result demonstrates again that our approach ensures computational efficiency and maintains moderate complexity, yet it still achieves accurate detection, confirming the effectiveness of our proposed fusion architecture.

**Figure 10:**
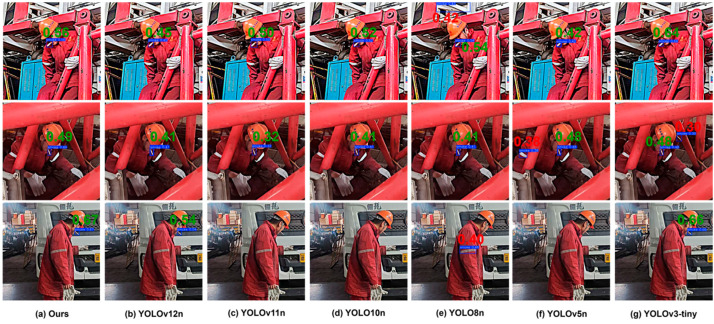
Representative field test results. Note: Green labels indicate correct detections, red labels indicate detection errors, and the absence of a label indicates missed detections.

In order to further demonstrate the generalization ability of the proposed model, real-world field tests have been conducted. Data were obtained using explosion-proof HD cameras made especially for oilfields. The experimental results demonstrate that the model performs effectively in actual situations and has strong generalization ability. Representative field test results are shown in Figure 10.

### 4.8. Validation of Performance Stability

We further examine the robustness of GCEA-YOLO by conducting three separate training runs with different random seeds (22,12,2025) on both the Multi-Angle Smoking Detection Dataset and the large-scale Smoking Detection Dataset. The results shown in Table 9 represent the average of these three runs. As can be seen from Table 9, GCEA-YOLO consistently outperforms the recently proposed baselines DAHD-YOLO and YOLO-AB across all metrics for both datasets. On the Multi-Angle Smoking Detection Dataset, it achieves an improvement of 8.8 percentage points in mAP@0.5 and 3.5 percentage points in mAP@0.5:0.95 compared to the better baseline DAHD-YOLO. On the Smoking Detection Dataset, the improvements amount to +3.6 percentage points for both mAP@0.5 and mAP@0.5:0.95, along with gains of +3.1 percentage points in precision and up to +2.7 percentage points in recall.

The fact that these stable and reproducible improvements hold true regardless of the random initialization and scale of the dataset demonstrates that the performance gain from GCEA-YOLO is genuine and not attributable to some special training condition or data regime. GCEA-YOLO achieves significantly better detection accuracy across various smoking detection scenarios, with only a modest increase in computational cost (7.8 GFLOPs and 3.4 M parameters vs. 6.4 GFLOPs and 2.6 M parameters for DAHD-YOLO).

## 5. Conclusions

To address the difficulties posed by complicated oilfield detection situations that need precise and timely outcomes, we propose GCEA-YOLO, an enhanced lightweight object detection model based on YOLOv11. We constructed a comprehensive training data set by collecting smoking behavior pictures under different environmental conditions, such as different lights, weather, and camera angles, so our model can work effectively in all kinds of situations. The model has four main components: ADown uses multiple path attention downsampling to preserve small target features but reduce spatial size, EfficientHead uses a lightweight structure to achieve real-time performance without affecting the accuracy of detection, and CSP-EDLAN uses two path convolutions to extract features in complex situations, which can obtain both local and global context information. GFPN uses multiscale deep learning to perform better when finding objects of different sizes, and it is effective for identifying distant objects or those hidden behind other objects. From the experiments, we can observe that GCEA-YOLO achieves significantly better performance compared with the YOLOv11n baseline: precision: 74.2% (improvement of 20.8%); recall: 55.5% (improvement of 6.9%); and mAP@0.5: 58.7% (improvement of 15.1%). Moreover, it only needs 1.4 GFLOPs more than the YOLOv11n baseline. These results demonstrate that GCEA-YOLO achieves an optimal balance of accuracy and efficiency, solving problems with small objects in challenging oilfields, and it meets the need for both precision and speed, thus providing valuable support for monitoring oilfields safely.

Although GCEA-YOLO has achieved good results, there are still some areas that need to be improved in the future. Firstly, as for the model lightweight optimization, although our current model can achieve comparable performance with an extra cost of 1.4 GFLOPs, it is still possible to utilize knowledge distillation, network pruning, and quantization-aware training [67] to further compress the model and reduce the amount of calculation required for deployment on resource-limited edge devices often found in oilfields. Secondly, with respect to improving the accuracy of the model, we can incorporate some advanced attention mechanisms (deformable attention and cross-scale attention) and new feature fusion methods to address extreme situations, such as dense smoke, heavy occlusion, and poor visibility. Thirdly, when it comes to the completeness of datasets, we currently have a dataset that could be substantially enhanced by adding different smoking poses, multiple people smoking at once, different ethnicities, and special cases such as e-cigarettes and people who are only partially visible. Also, using videos instead of pictures and marking finer details, such as the stage of smoking or the type of cigarettes, etc., can improve the system’s understanding of actions. And performing cross-domain validations on other kinds of safety-critical detections within industrial environments would also provide insight about how well this model generalizes and whether it is applicable outside the oilfield smoking detection context.

Operational and Ethical Considerations: While GCEA-YOLO demonstrates good technical performance, several considerations must be addressed before widespread industrial deployment. Operationally, deploying deep learning models in oilfield environments requires robust infrastructure for real-time processing, reliable network connectivity in remote locations, and comprehensive maintenance procedures for harsh conditions (extreme temperatures, dust, and vibrations). False positives may lead to unnecessary interventions and worker frustration, while false negatives could overlook safety violations; therefore, thresholds must be carefully tuned and monitored by humans. Ethically, continuous video surveillance raises privacy concerns regarding worker monitoring, data storage, and potential misuse. Clear policies on data retention, access control, and transparency are essential for keeping workers informed and protecting their rights. The system should serve as a safety assistance tool rather than a punitive mechanism, with human review procedures to prevent automated decision-making that could unfairly impact employment. Deployment must comply with local labor laws, privacy regulations, and ethical guidelines. Stakeholder consultation involving workers, management, and ethics committees is essential to ensure responsible implementation that balances safety objectives with individual rights and dignity.

## Figures and Tables

**Table 1:** Comparison of recent specialized smoking detection models with respect to the three unresolved bottlenecks.

Method	Year	Main Improvement	Aggressive Downsampling Loss	Cross-Scale Fusion	Lightweight Head
Fu et al. [12]	2020	Dual-branch hand + smoke	Not addressed	Partial	Not addressed
Wang et al. [37]	2021	SD attention + gradient balancing	Not addressed	Addressed	Not addressed
Wang et al. [38]	2019	CARAFE feature reshaping	Not addressed	Addressed	Not addressed
Zhang et al. [39]	2025	ECA-FPN	Not addressed	Addressed	Partial
Zhang et al. [39]	2025	DAHD-YOLO	Not addressed	Addressed	Partial

**Table 2:** Detailed architecture and training configuration of GCEA-YOLO.

Part	Stage	Layers	Channels	Feature Map Size	Description
BACKBONE	Input	0–1	64 → 128	H/2 → H/4	Initial downsampling with Conv layers
	Stage 1	2	256	H/4 × W/4	CSP-EDLAN feature extraction
	Stage 2	3–4	256 → 512	H/8 × W/8	ADown + CSP-EDLAN
	Stage 3	5–6	512	H/16 × W/16	ADown + CSP-EDLAN
	Stage 4	7–8	1024	H/32 × W/32	ADown + CSP-EDLAN
	Neck	9–10	1024	H/32 × W/32	SPPF + C2PSA for multiscale aggregation
HEAD	P5Branch	11–14	512 → 1024 → 512	H/32 × W/32	Top–down pathway initialization
	P4Branch	15–19	512	H/16 × W/16	Multiscale fusion with upsampling
	P3Feature	20–23	256	H/8 × W/8 (80 × 80)	Small-object features (P3/8)
	P4Feature	24–27	512	H/16 × W/16 (40 × 40)	Medium object features (P4/16)
	P5Feature	28–32	1024	H/32 × W/32 (20 × 20)	Large object features (P5/32)
DETECTION	Detect Head	33	1 × 3	Multiscale (P3 + P4 + P5)	Efficient detection with 8400 predictions
Training Configuration	Input size	-	-	640 × 640	Multiscale range [480,800] during training
	Head type	-	Detect_Efficient	Anchor-free	3 scales, direct prediction
	Loss	-	CIoU + DFL + BCE	-	λ_box = 0.05, λ_cls = 0.5, λ_dfl = 1.0
	Assigner	-	TaskAlignedAssigner	top-k = 10, α = 0.5, β = 6.0	-

**Table 3:** Configuration parameters.

Hardware	Software
CPU: Intel i9-12,900K	Windows11 Version 23H2
GPU: NVIDIA GeForce RTX 4090D	Python 3.11.11
Video memory: 24 GB GDDR6X	PyTorch 2.0.0
Memory: 2 × 32 DDR4	CUDA 11.8

**Table 4:** Ablation experiment results. Note: √ indicates the inclusion of the corresponding module; × indicates its exclusion.

ADown	CSP-EDLAN	EfficientHead	GFPN	Precision	Recall	mAP@0.5	mAP@0.5:0.95	FPS	Flops/G	Params/M
×	×	×	×	0.614	0.519	0.51	0.218	232.0	6.4	2.6
√	×	×	×	0.701	0.491	0.517	0.224	206.1	5.4	2.1
×	√	×	×	0.656	0.516	0.534	0.232	259.0	6.9	2.4
×	×	√	×	0.649	0.531	0.523	0.223	253.0	5.1	2.3
×	×	×	√	0.635	0.504	0.538	0.239	176.3	8.2	3.7
√	√	×	×	0.703	0.525	0.543	0.222	229.7	5.9	2.0
√	×	√	×	0.680	0.484	0.539	0.237	235.2	4.1	1.8
√	×	×	√	0.694	0.511	0.540	0.225	177.8	7.4	3.3
×	√	√	×	0.695	0.521	0.539	0.236	274.3	5.7	2.2
×	√	×	√	0.630	0.494	0.530	0.239	169.8	10.3	4.2
×	×	√	√	0.741	0.465	0.528	0.228	207.8	7.2	3.5
√	√	√	×	0.711	0.545	0.565	0.239	249.3	5.9	2.0
√	√	×	√	0.686	0.530	0.569	0.235	162.8	9.0	3.7
√	×	√	√	0.671	0.521	0.545	0.234	184.4	6.2	3.0
×	√	√	√	0.703	0.511	0.534	0.240	176.3	9.1	3.9
√	√	√	√	0.742	0.555	0.587	0.255	181.9	7.8	3.4

**Table 5:** Performance comparison of different downsampling modules in GCEA-YOLO.

Attention Module	*p*	R	mAP@0.5	mAP@0.5:0.95	Flops/G	Params/M
SPDConv [43]	0.730	0.504	0.556	0.232	13.8	6.6
MixDown [46]	0.684	0.533	0.563	0.246	9.7	3.7
HWD [61]	0.697	0.550	0.557	0.236	8.1	3.6
LDConv [62]	0.603	0.384	0.398	0.159	7.9	3.5
ContextGuidedDown [63]	0.701	0.533	0.557	0.235	12.0	5.3
Adown [19]	0.742	0.555	0.587	0.255	7.8	3.4

**Table 6:** Comparison of different methods and their performance on the Multi-Angle Smoking Detection Dataset.

Model	*p*	R	mAP@0.5	mAP@0.5:0.95	FPS	Flops/G	Params/M
Faster-RCNN [28]	0.73	0.56	0.639	0.275	104.70	37.52	28.3
SSD [31]	0.618	0.467	0.535	0.225	187.55	30.43	23.7
YOLOv3-tiny [25]	0.603	0.45	0.440	0.164	719.6	18.9	12.1
YOLOv5 [64]	0.636	0.421	0.437	0.162	313.8	7.2	2.5
YOLOv8 [65]	0.616	0.450	0.442	0.175	321.8	8.2	3.0
YOLOv10n [61]	0.615	0.428	0.399	0.150	115.6	8.4	2.7
YOLOv11n [33]	0.614	0.519	0.510	0.218	232.0	6.4	2.6
YOLOv12n	0.630	0.491	0.500	0.215	161.7	5.8	2.5
GCEA-YOLO	0.742	0.555	0.587	0.255	181.9	7.8	3.4

**Table 7:** Comparison of different methods and their performance on the Smoking Detection Dataset.

Model	*p*	R	mAP@0.5	mAP@0.5:0.95	Flops/G	Params/M
Faster-RCNN [28]	0.853	0.744	0.805	0.409	37.52	28.3
SSD [31]	0.830	0.638	0.734	0.391	30.43	23.7
YOLOv3-tiny [25]	0.829	0.69	0.737	0.374	18.9	12.1
YOLOv5 [64]	0.799	0.642	0.722	0.368	7.2	2.5
YOLOv8 [65]	0.777	0.677	0.740	0.394	8.2	3.0
YOLOv10n [61]	0.793	0.657	0.738	0.390	8.4	2.7
YOLOv11n [33]	0.868	0.752	0.851	0.511	6.4	2.6
YOLOv12n	0.867	0.769	0.866	0.525	5.8	2.5
GCEA-YOLO	0.883	0.796	0.888	0.545	7.8	3.4

**Table 8:** Comparison of different methods and their performance on COCO2017.

Model	*p*	R	mAP@0.5	mAP@0.5:0.95
YOLOv11n [33]	0.448	0.303	0.301	0.197
GCEA-YOLO	0.459	0.324	0.326	0.214

**Table 9:** Robustness evaluation of GCEA-YOLO across different random seeds on two Smoking Detection Datasets.

Datasets	Model	*p*	R	mAP@0.5	mAP@0.5:0.95	FPS	Flops/G	Params/M
Multi-Angle Smoking Detection Dataset	DAHD-YOLO [39]	0.663	0.496	0.499	0.220	187.1	6.4	2.6
	YOLO-AB [66]	0.640	0.494	0.516	0.219	188.5	7.5	2.7
	GCEA-YOLO	0.742	0.555	0.587	0.255	181.9	7.8	3.4
Smoking Detection Dataset	DAHD-YOLO [39]	0.857	0.769	0.853	0.510	152.7	6.4	2.6
	YOLO-AB [66]	0.878	0.745	0.852	0.516	144.6	7.5	2.7
	GCEA-YOLO	0.883	0.796	0.888	0.545	140.9	7.8	3.4

## Data Availability

The smoking behavior detection dataset and trained models presented in this study are available upon request from the corresponding author. The data are not publicly available due to privacy and safety considerations in oilfield operations.
